# Male Fertility in Spondyloarthritis: from Clinical Issues to Cytokines Milieu. A Narrative Review

**DOI:** 10.1007/s11926-024-01153-w

**Published:** 2024-06-20

**Authors:** Silvia Scriffignano, Fabio Massimo Perrotta, Ennio Lubrano

**Affiliations:** Academic Rheumatology Unit, Dipartimento Di Medicina E Scienze, Della Salute “Vincenzo Tiberio”, Università Degli Studi del Molise, Via Giovanni Paolo II, C/da Tappino, 86100 Campobasso, Italy

**Keywords:** Spondyloarthritis, Ankylosing spondylitis, Psoriatic arthritis, Male fertility, Semen quality, Sperm quality

## Abstract

**Purposeof Review:**

Male fertility is an emergent issue that should be considered in clinical practice, when dealing with chronic inflammatory diseases in young men. As it is known, the chronic inflammation is the main pathophysiologic mechanism in some rheumatological conditions such as spondyloarthritis (SpA), Ankylosing Spondylitis (AS) and Psoriatic Arthritis (PsA). Therefore, it is paramount to be aware if these diseases could impair male fertility, both due to the inflammation or to the treatments needed: we reviewed the literature on the most relevant and recent evidence on male fertility in patients affected by SpA, AS and PsA.

**Recent Findings:**

Rheumatological inflammatory diseases (included SpA, AS and PsA) could impair the family planning in man life, especially when diagnosed at young age. Moreover, focusing on sperm quality, it seems that a link between sperm quality impairment and a higher disease activity exist.

Focusing on therapies, Tumor Necrosis Factor inhibitors showed a safety profile on human male fertility in clinical studies. Recently, a prospective study and two double-blind placebo-controlled trials assessed the impact of methotrexate and Filgotinib on semen parameters, respectively, showing a safety profile of these drugs on human semen quality. However, there are no clinical data on the impact of Interleukin (IL)17 inhibitors(i), IL12-23i and IL23i.

**Summary:**

Concerning male fertility in SpA, AS and PsA, an unmet clinical need is still present and new studies are needed to understand the association between these diseases and male fertility, and the implication of the therapies used for these diseases. This narrative review provides an overview of the available data on male fertility in patients affected by SpA, AS and PsA.

## Introduction

Male fertility is an emergent issue that should be considered in clinical practice, especially when a chronic inflammatory disease is diagnosed to a man in his youth [1]. In fact, inflammation, through the cytokines release, reactive oxygen species (ROS) production and oxidative stress promotion, could impair male fertility [1]. Systemic inflammation, as well as urogenital tract inflammation, play a role in the male fertility impairment [2]. It was described a higher risk of infertility both in male with acute inflammation in the urogenital tract (due to infections) [2] and in some chronic diseases, such as ulcerative colitis [3], obesity [4] and diabetes [5].

Chronic inflammation is the main pathophysiologic mechanism in some rheumatological conditions [6], such as Spondyloarthritis (SpA), Ankylosing Spondylitis (AS) and Psoriatic Arthritis (PsA), that are usually diagnosed in young/adult men, during their peak reproductivity years [6]. Therefore, for these patients, it is not of secondary importance to be aware of the potential impartment of their fertility due to both the disease and the treatments they need [7••, 8].

In this scenario, the two sides of the coin could be: the impact of the disease on male fertility (for example: “how does SpA/AS/PsA disease activity impair male fertility?”) and, on the other side, the influence of the treatments used for these conditions on male fertility.

In this narrative review, we try to analyze the most relevant evidence on male fertility in patients affected by SpA, AS and PsA.

Moreover, the possible impact on male fertility of the newest drugs used for SpA, including AS and PsA, namely biologic Disease Modifying Anti-Rheumatic Drugs (bDMARDs) and target synthetic (ts) DMARDs was discussed.

## Methods

We searched PubMed and the Cochrane library for articles and reviews in the English language published in the last 25 years (between 1st Jan 1997 and 31st August 2023). The searched words were: “spondyloarthritis”; “ankylosing spondylitis”, psoriatic arthritis”, “male fertility”, “male infertility”, “fertility impairment”, “sperm/semen quality”, “varicocele”, “follicle-stimulating hormone", “inhibin B”, “biological and target synthetic disease modified anti rheumatic drugs”, “tumor necrosis factor inhibitor”, “interleukin 17 inhibitors”, “interleukin12-23 inhibitors”, “interleukin 23 inhibitors”, “Janus Kinase inhibitors”. The research was limited to human clinical studies, clinical trials, clinical cases, reviews, and meta-analyses. We followed the guidelines for writing a narrative review previously published [9]. We also added other papers suggested by co-authors to improve the quality of the review.

## Definition of Infertility

Infertility is defined as the inability of a couple to conceive even after one year of unprotected, frequent sexual intercourse [10]. The male is solely responsible in about 20% of cases and he is a contributor in another 30%-40% of all infertility cases [11].

**Fig. 1:**
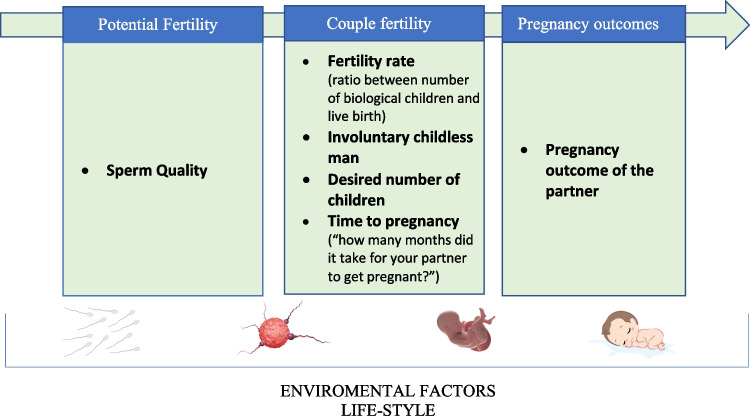
The different phases, in which male fertility may be assessed

Generally, male fertility is assessed by using several outcomes and based on the time in which it is evaluated we can schematically distinguish three different phases: “potential fertility”, “couple fertility” and “pregnancy outcome of partners” (Fig. 1). In the first phase the male fertility is independent by the female (for example assessed by the semen/sperm analysis), instead in the others two phases, factors linked to male, and female contribute (Fig. 1).

After briefly clearing up the definition of infertility, following we described the most relevant studies on this topic, analysing the “disease contribution” and the “treatment contribution” in fertility impairment in patient affected by SpA, AS and PsA.

Moreover, the possible link between some key cytokines in SpA, AS and PsA pathogenesis and male fertility is also discussed.

## Male Fertility in Spondyloarthritis

The effects of the autoimmune rheumatological diseases on male reproductive health were recently summarized in a systematic review: the authors paved the way towards the need to characterize the “male fertility profile” for each rheumatological disease and the need of prospective large studies [7••]. Concerning SpA, they included eight studies (two cross sectional and six case–control studies) from which the results are not always concordant: in some studies, the disease activity seemed not impairing the male semen parameters, while in other studies it was reported an impaired semen quality in those patients with a higher disease activity [7••]. Moreover, it has been described a high rate of varicocele in SpA patients [7••]: the explanation of this phenomenon is probably the consequence linked to a frequency increase of the Valsalva’s maneuver that SpA patients implement due to the secondary paravertebral muscles’ weakness (due to the chronic back pain). It causes an increase of the abdominal pressure that could impair the spermatic vein valves and therefore could be linked to a higher varicocele risk [12].

The larger study on the impact that rheumatological diseases have on male fertility is a recent Dutch retrospective multicenter-study (iFAME-fertility study), in which it was assessed the impact of inflammatory arthritis on male fertility [13•] and in a following study of the same cohort, the pregnancy outcomes of partners of men diagnosed with inflammatory arthritis [14•].

The male patients included in these studies were 40 years old or older (men that completed their family planning) and had a rheumatological diagnosis of inflammatory arthritis (rheumatoid arthritis (RA), juvenile idiopathic arthritis, AS, PsA, reactive arthritis or enteropathic arthritis).

In the first study [13•], the data of 628 patients, of which 320 were SpA patients (including PsA), were analyzed and stratified in three groups according with the age at the time of inflammatory arthritis diagnosis: ≤ 30 years, between 31–40 years and ≥ 41 years. Due to the retrospective nature of this study, for each group, the assessed outcomes were:total number of biological children (fertility rate), total number of pregnancies per man, childlessness, desired number of children, need of medical evaluation for fertility problems and time to pregnancy.

As emerging result from this analysis, the inflammatory arthritis diagnosis before 30 years or between 31–40 years was associated with a lower fertility rate when compared either with those patients with an inflammatory arthritis diagnosis after 40 years, or the general population, resulting in a significative lower number of biological children for those SpA patients that received diagnosis before 41 years old.

In the same wavelength, among men diagnosed ≤ 30 years, there was a lower total number of pregnancies, and the involuntary childless men were more numerous rather than men diagnosed between 31 and 40 years and ≥ 41 years. Even if in this study the specific therapies of patients were not mentioned, it was showed that patients with inflammatory arthritis diagnosis before 41 years were concerned about medications, as a possible factor that harmed their child.

In the followed study of the same cohort [14•], it was described the pregnancy outcomes of the partners of men diagnosed with inflammatory arthritis (408 patients included), using a self-reported questionnaire: a noteworthy result was the difference in the percentage of miscarriage between the pregnancies conceived after or before the diagnosis of inflammatory arthritis. The rate of miscarriage was statistically higher in pregnancies conceived after inflammatory arthritis diagnosis (12.27%) compared with pregnancies conceived before the diagnosis (7.53%); this result was confirmed after adjustment for other confounding factors [odd ratio 2.03 (CI 95% 1.12–3.69)] [14•].

Both these studies, considering all the limitations of the retrospective design, are nowadays the largest ones on this topic and they undoubtedly help the physicians to consider the covered importance of the male fertility when dealing with young patients affected by SpA.

## Sperm Quality in SpA, AS and PsA

A crucial point in male fertility is the sperm/semen quality [14•].

There are few studies that assessed the change in semen parameters of SpA patients related to disease activity and as compared to healthy control; however, all these studies are characterized by a small sample size.

A case–control study showed the sperm analysis differences of 10 SpA patients (of whom 5 PsA) accordingly with their disease activity, before and after they started any rheumatological therapy [15].

In this case, higher disease activity indices [Bath Ankylosing Spondylitis Disease Activity Index (BASDAI) and Disease Activity Score 28 (DAS-28)] negatively influence sperm parameters with a decrease of the progressive motility in sperm analysis when disease activity was higher. In fact, semen analysis was performed in two different time points: before they started therapy and after one year of therapy with Tumor Necrosis Factor inhibitors (TNFi) (adalimumab). In this case, TNFi do not appear to damage testicular function or spermatogenesis, indeed it showed a trend toward the improvement of sperm parameters [15]. Moreover, the results of the comparison of sperm parameters between patients before they started treatment and healthy controls proved a significant decrease of the percentage of progressive motility in patient’s vs controls [15]. Therefore, from these results it seems that disease activity could alter some sperm parameters. This result was also confirmed by another case–control study [16], in which the sperm quality of 20 SpA patients treated with TNFi (adalimumab or etanercept or infliximab) (median BASDAI of 1.5) was compared with those of 11 SpA patients without TNFi therapy (median BASDAI 4.38). SpA patients without TNFi therapy had a poorer sperm motility and vitality [16]. Moreover, the spermiograms of all patients receiving TNFi treatment were compared with those from 102 healthy controls and no significant differences were found. On the other hand, the 11 SpA patients without TNFi treatment, compared with the healthy control group, showed a significant reduction in sperm motility and vitality [16].

Instead, another published study diverges from these results concerning the impact of the disease activity on sperm parameters but confirm the safety profile of TNFi on semen quality [17]. Namely, the authors compared the sperm quality of 23 patients affected by active AS with healthy control: the sperm analysis did not differ between the groups, showing that the high disease activity, in this case, did not affect semen quality. However, accordingly with the previous studies, a sub-analysis of the same group showed a great safety profile of TNFi on semen quality both for short (3–6 months) and long (12 months) term treatment period [17].

Regarding treatment and semen quality, the pooled results of a two phase 2 randomized doble-blind placebo-controlled trials, MANTA and MANTA-Ray, were recently published [18••]. These trials were designed to determine the impact of Filgotinib on human semen parameter. Enrolled patients were affected by moderate/severe active inflammatory bowel disease in MANTA trial and by active RA, AS, PsA and non-radiographic SpA in MANTA-Ray trial [18••]. The primary endpoint in both trials was the assessment of the proportion of participants with a ≥ 50% decrease from baseline in sperm concentration after 13 weeks of treatment (Filgotinib vs placebo): this reduction was reported for the 6.7% of patients after 13 weeks of Filgotinib treatment and for the 8.3% of patients after placebo treatment. This result together with the secondary aims of these trials support the absence of measurable impact of Filgotinib (13 weeks) on human semen parameters [18••].

Another recent study (iFAME-MTX) [19••] assessed prospectively the testicular toxicity profile of Methotrexate (MTX) on male fertility assessing semen parameters, reproductive endocrine axis, and sperm DNA fragmentation index (a novel outcome to assess the integrity or damage of sperm DNA). Included patient (20 patients affected by RA, PsA, SpA and psoriasis) were assessed before they started MTX and after 13 weeks of MTX treatment (mean MTX dose: 16 mg/week): semen parameters and sperm morphology did not statistically change before and after MTX treatment. Moreover, the median value of the sperm DNA fragmentation index was higher in the pre MTX-exposure samples compared with the post exposure sample, even if this difference was not statistically significant different, meaning a possible higher percentage of DNA damage in those patients with rheumatological disease before they started MTX treatment (and therefore with more active disease).

Even if the role of SpA activity is not completely defined, it seems that a higher disease activity could impair semen quality; however, all the assessed studies confirmed a safety profile of TNFi concerning semen quality of SpA patients [7••, 15–17], even if a prospective large study/trial is missing for this pharmacological class. Instead, the most recent studies MANTA/MANTA-Ray trials [18••] and iFAME-MTX study [19••] showed the possibility to use a new tsDMARD, namely Filgotinib and an established csDMARDs, namely MTX, in male patient affected by SpA without any concern on their fertility (assessed based on sperm parameters).

## Varicocele in SpA, AS and PsA

Although semen analysis is the first step to study male infertility, another condition, linked to male infertility, is varicocele [20].

It should be assessed, not only for its link with male fertility impairment, but also because its incidence (clinically and on color doppler examination) is increased in AS patients compared with age-matched control [12].

In a large population-based case–control study, on the Taiwan’s National Health Insurance Research Database, a higher varicocele risk in AS patients was confirmed (odd ratio 1.46 (95% CI 1.18–1.81) [21].

In a comparison study between 20 AS patients with 24 healthy controls, the varicocele prevalence was higher in AS patients (40% vs 8%, *p* = 0.027), however, in this case, sperm quality did not reveal statistically significant differences between AS patients and healthy controls [22]. Therefore, in this case, sperm quality was not impaired in those patients with varicocele. On the same wavelength in another study the comparison between AS patients with varicocele and AS patients without varicocele did not show any difference in anti-sperm antibodies, hormones, inflammatory markers, and disease activity scores [23].

Therefore, varicocele clinical relevance needs to be evaluated, to understand if it may contribute to infertility in AS, and more in general in SpA and PsA patients.

As mentioned above, the reasons under a higher prevalence of varicocele in AS could be related to a higher abdominal pressure [12], but to clarify this point new studies are needed; moreover, nowadays there are no studies on varicocele in PsA patients.

## Hormones that Drive Male Fertility in SpA, AS and PsA

Another aspect that should be considered is the hormone profile linked to male infertility: in particular, inhibin B is a glycoprotein hormone produced by Sertoli testicular cells, that regulates the Follicle-Stimulating Hormone (FSH) level throughout a negative feedback mechanism [24]. Its serum levels are strongly positively correlated with testicular volume and sperm counts. Generally, in infertile patients, inhibin B decreases and FSH increases [24].

The number of studies that assess the sex endocrine hormone profile of SpA patients are very limited and the results are not always comparable due to the heterogeneity of these studies.

In a case control study, the inhibin B and FSH serum level were compared between 20 AS patients (moderate/high disease activity) and 24 healthy controls: no differences between these two groups were found [25]. Therefore, this study seems to support the hypothesis that the hormone axis of AS patients with an active disease is like in healthy subjects.

Differently from this result, in the iFAME-MTX study [19••] the authors studied the differences on the reproductive endocrine axis (testosterone, sex hormone binding globulin, luteinizing hormone, FSH, inhibin B) between MTX-naïve pre-exposure patients, MTX-naïve post-exposure patients and healthy controls. The only difference found was on inhibin B: the patients, both before they started treatment and after treatment exposure, had a lower median inhibin B serum concentration compared to healthy controls [19••]. Therefore, in this case the final message is that patients affected by RA, PsA, SpA and psoriasis had a lower concentration of serum inhibin B level compared with healthy controls [19••].

Moreover, in MANTA and MANTA-Ray trial the comparison of the reproductive endocrine axis did not show any change before and after treatment with Filgotinib [18••].

Considering the very poor and heterogeneous data on the sex hormone endocrine axis study in SpA patients, new studies are needed to find more answers on this topic and to understand their impact on male fertility.

## Cytokines and Therapeutic Complaints on Male Fertility in SpA, AS and PsA

The semen of healthy fertile men contains a broad array of immunologic factors, such as cytokine and immunoglobulins. Normal values for these immunological factors were proposed [26], showing a low-level concentration for some soluble factors such as Interleukin (IL) 17 and TNFα [26].

**Fig. 2:**
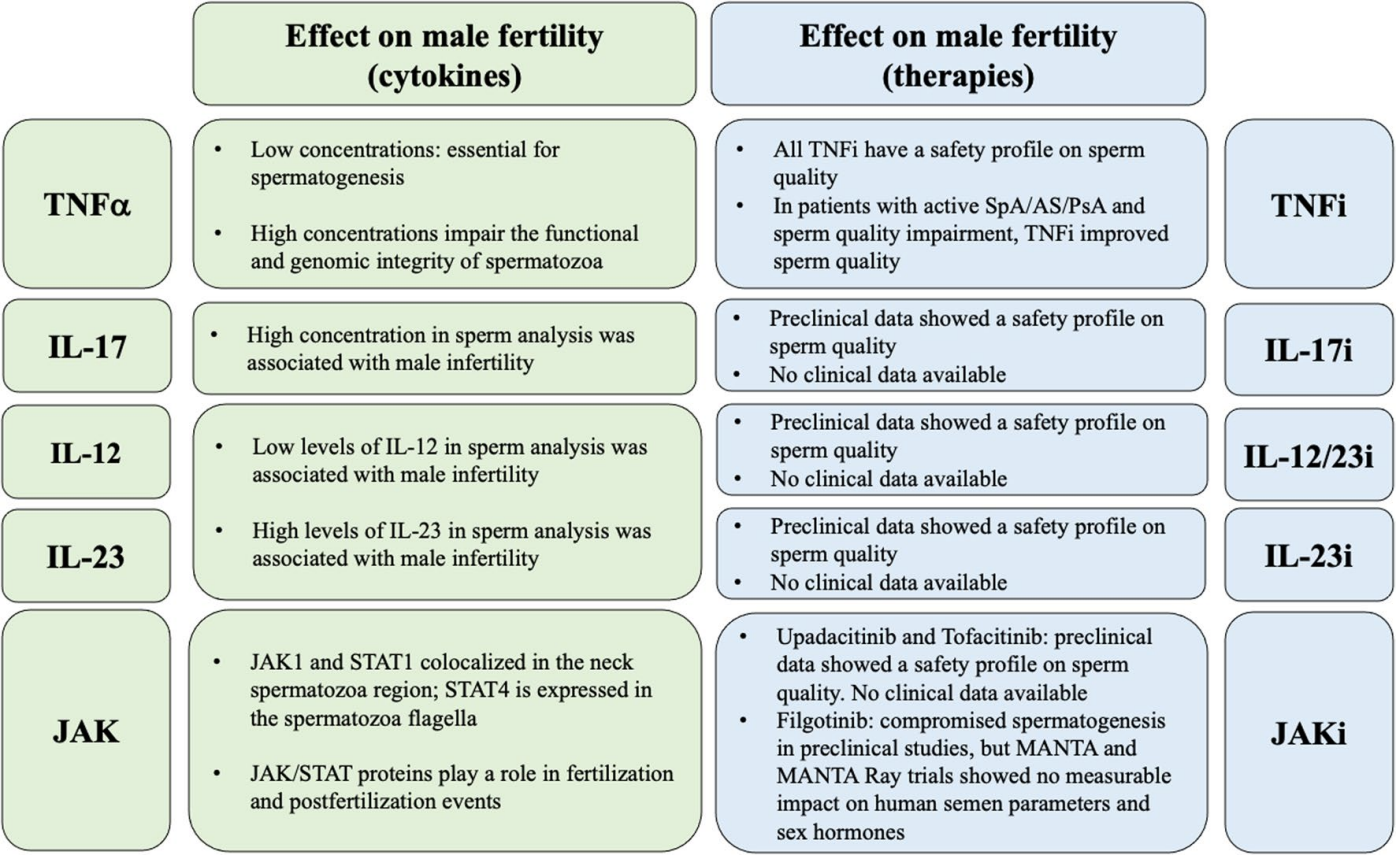
The effect of some key SpA, AS and PsA cytokines, when assessed in sperm analysis and the effects that the target therapies for those cytokines have on sperm quality in those patients. *IL* Interleukin *IL-17i* interleukin 17 inhibitors, *IL-12/23i* interleukin 12–23 inhibitors, *IL-23i* interleukin 23 inhibitors, *JAK* Janus Kinase, *JAKi* Janus Kinase inhibitors, *STAT* Signal Transducer and Activator of Transcription, *TNF* Tumor Necrosis Factor, *TNFi* Tumor Necrosis Factor inhibitors

Following, we described the interplay of some crucial cytokines in the pathogenesis of SpA, AS, PsA with male fertility, focusing on how their pharmacological inhibition could impact on male fertility (Fig. 2).

## Role of TNFi

In human testis, TNFα is physiologically produced, acting a role in the regulation of spermatogenesis, with a down regulation of Fas ligand (an inductor of testicular apoptosis) [27]. Therefore, TNFα, in low concentrations, is essential for the spermatogenesis [27].

However, spermatozoa exposed (in vitro) to high/pathological concentrations of TNFα significantly loss their functional and genomic integrity; in fact, spermatozoa quality declined following incubation with TNFα in a dose-dependent and time-dependent manner [28].

There are different studies supporting the evidence of the association between TNFα gene polymorphisms, that cause an overexpression of TNFα, and male infertility risk [29–31].

TNFα is a key cytokine in the pathogenetic process of SpA, AS and PsA [6, 32], in fact different bDMARDs target the TNFα are widely used [32]: infliximab, etanercept, adalimumab, golimumab, certolizumab. This pharmacological class was the first one, as bDMARDs, used for SpA, AS and PsA, therefore the number of studies on these therapies are numerically larger compared with other bDMARDs or tsDMARDs.

As mentioned above, in general, the exposure to each TNFi therapies appears to be safe on sperm quality, indeed some studies showed an improvement of sperm quality in those patients with an active disease after they started TNFi therapy [15, 16].

It was studied the effect on sperm quality of the pharmacological TNFα neutralization: infliximab, on TNFα exposed spermatozoa, improves (in vitro) the sperm motility and membrane integrity [28].

Clinical data, supporting this in vitro evidence come from a small Greek study, in which the pregnancy outcome of 10 patients (7 AS and 3 PsA) after paternal exposure to infliximab showed a favorable course [33].

A phase 1 study on certolizumab pegol and sperm quality showed no treatment effects on sperm quality in healthy males treated with a single injection of 400 mg compared to placebo [34].

On the other hand, in a case series of 3 AS patients treated with infliximab the presence of asthenoazoospermia (in two of these patients) was described [35]. However, as the authors commented, they were unable to prove that the TNFi therapy was directly linked to asthenoazoospermia.

Overall, all data seem to confirm the “secure profile” of the TNFi on the sperm quality and male fertility. It is also supported by the last guideline of the American College of Rheumatology (ACR) for the management of reproductive health in rheumatic and musculoskeletal diseases, in which it is strongly recommended to continue therapy with all TNFi for those men who are planning to be father of a child [36].

Likewise, the British Society of Rheumatology in the last guideline on the prescribing drugs in pregnancy and breastfeeding recommend the TNFi paternal exposure as compatible with pregnancy (GRADE 1C, strength of agreement 99.3%) [37].

All these data support the hypothesis of a possible link between TNFα and male fertility: higher levels could impair semen quality and on the other hand the use of all treatment that target this cytokine showed a safety profile on male fertility.

## Role of IL17i

Even IL-17 seems to play a role in male infertility [38]: in a recent case–control study, a higher concentration of IL-17 was found in male diagnosed as infertile ones (based on the semen analysis), when compared with healthy male (with normal semen analysis) [39].

The mechanism throughout IL-17 impairs fertility is not completely understood, even if new evidence showed that IL-17A, per se, could impair sperm motility and decreases viability by triggering increased mitochondrial ROS production and inducing sperm apoptosis [38].

Moreover, comparing the cytokine semen profile, between infertile patients affected by chronic urethroprostatitis and fertile patients with the same disease, IL-17 levels were higher in infertile patients [40].

IL-17 is a pivotal cytokine in SpA, AS and PsA [41], and nowadays there are specific bDMARDs therapies targeted IL-17 (secukinumab, ixekizumab, bimekizumab) [42].

Unfortunately, there are not studies focused on IL-17 levels in semen specimens coming from SpA, AS and PsA patients and on the effects of the IL-17i on human male fertility.

However, in preclinical studies, secukinumab [43], ixekizumab [44] and bimekizumab [45] showed a safety profile in terms of male fertility in animal models.

The unmet need on this topic should be covered due to the awareness of the IL-17 pathogenetic role in SpA, the possible link of IL-17 with male infertility and the ever-wider use of IL17i in these diseases.

## Role of IL 12-23i and IL 23i

IL-12 and IL-23 are involved in male fertility: lower level of IL-12 and higher level of IL-23 were associated with male fertility impairment [46].

Both these two cytokines have a key role in the pathogenesis of SpA, and in particular in PsA [47]. In fact, there are three monoclonal antibodies, ustekinumab, guselkumab and risankizumab, that target respectively the p40 subunit of the IL-12 and IL-23 (the first one) and the p19 subunit of the IL-23 (the second and third ones), approved and used for PsA [48, 49].

There are no studies focused on these cytokines in human sperm of patients affected by SpA, AS and PsA.

In preclinical studies ustekinumab [50], guselkumab [51] ad risankizumab [52] showed a safety profile on male fertility in animal models.

As suggested for IL-17, the unmet need on this topic should be covered due to the awareness of the pathogenetic role of IL-12 and IL-23 in SpA and in particular in PsA, and the ever-wider use of IL12-23i and IL23i in this disease.

## Role of JAKi

Beyond the role of Janus kinase as a signal transducer activator of transcription (JAK/STAT) and as a transcription factors, it was shown that JAK1 and STAT1 colocalized in the neck spermatozoa region and that STAT4 is expressed in the spermatozoa flagella [53]. The localization in sperm structural component suggests a possible different role from their well-known transcription factor activity in somatic cells, but further investigations are required to determine their role in sperm function [53]. Moreover, the inducers of sperm acrosome reaction variably modulated the phosphorylation of STAT 4. These data are indicative of an important role for the spermatozoa JAK/STAT proteins in fertilization and post-fertilization events [54].

A recent study on patients affected by azoospermia showed a possible link between azoospermia and JAK2 expression. In fact, a lower expression of JAK2 mRNA and high expression of miR-135a-5p (a micro-RNA able to inhibits JAK2 expression) were related to asthenozoospermia and male infertility [55].

Since JAK-STAT pathway is crucial in the pathogenesis of SpA, AS and PsA, some therapies able to modulate these transductions are recently adopted for the treatment of these diseases, namely Tofacitinib and Upadacitinib [56]. Both these two molecules are able to reduce the production of different cytokines by the inhibitions of the JAK-STAT signal. Preclinical studies on animal models showed a safety profile both for Tofacitinib [57] and Upadacitinib [58] on male fertility. Moreover, in a recent study, the pregnancy outcome of partners of male (affected by ulcerative colitis), exposed to Tofacitinib before the conception, were assessed. Fourteen cases of paternal exposure to Tofacitinib (doses of 5 mg or 10 mg twice daily) were analyzed, showing pregnancy outcome similar for those of the general populations [59]. Nowadays, the effect of Tofacitinib and Upadacitinib on male fertility in patients affected by SpA, AS and PsA is unknown, even if preclinical data and data from patients with ulcerative colitis are reassuring.

The only JAKi that showed a possible impairment in male fertility in preclinical studies was Filgotinib, recently studied for AS and PsA [60, 61]. Due to these preclinical results, the MANTA and MANTA-Ray trials have been recently concluded: 2 randomized double-blind placebo-controlled trial to assess the impact of Filgotinib on semen parameters and sex hormones, in patients affected by inflammatory bowel diseases and inflammatory arthritis respectively [18••].

As mentioned above, the pooled results from these two trials reassuring the physicians on the consequences of the Filgotinib use on male fertility, in fact, no measurable impact on semen parameters and sex hormones was found in those patients treated with Filgotinib for at least 13 weeks [18••].

These two trials are the first large-scale clinical trials measuring the effect of a treatment on sperm in men with rheumatological diseases. It is desirable that this kind of clinical trial will be plan for other kinds of therapies.

## Discussion

In this narrative review we summarize the evidence on male fertility in patients affected by SpA, AS and PsA.

Some studies [15, 16] suggest a crucial role of SpA/AS/PSA activity as contributor for male fertility impairment, but this concept is not always confirmed [17]. These discordant results could be linked to the difficulty to detect the influence of a single factor in a multifactorial process like infertility, other than the small sample size.

Regarding the treatment’s effects on male fertility, there are different case–control and cross-sectional studies that support a safety profile of TNFi on semen analysis [15–17], a prospective study on MTX [19••] and 2 clinical trials on Filgotinib [18••] that showed the absence of semen impairment in patients affected by inflammatory arthritis that use these drugs.

The i-FAME studies [13•, 14•], are the largest studies that assess the impact that inflammatory arthritis have on male fertility. These studies support the male fertility impairment risk in those patients receiving SpA diagnosis in young age and a higher number of miscarriages for those pregnancies conceived after inflammatory arthritis diagnosis. These results should rise the attention bare when dealing with SpA patients, but it is important to note that both the studies are focused on outcomes measures linked to the couple fertility, such as the total number of biological children, the total number of pregnancies per man, time to pregnancy, the pregnancy outcomes (for example miscarriage), therefore it is very difficult to define the precise “male contribution” in these results.

Moreover, other factors like smoke and alcohol intake, known for their impact on male fertility [62, 63] were considered only for the iFAME study on pregnancy outcomes [14•] but not for the other iFAME study [13•].

Furthermore, beyond smoke and alcohol, other environmental factors and lifestyle showed a crucial role in male fertility [64, 65]: they are not included in any study that assess male fertility in rheumatic patients, therefore it could be useful include those factors in all future studies that assess male fertility.

All these aspects underline the complexity to identify the best fertility outcome measure and the need to consider a lot of other confounders factors.

Among outcome measure, the cornerstone in the assessment of male fertility is the sperm/semen quality analysis. The “least common denominator” in all studies that assessed the sperm quality in SpA is the small sample size, except for the recent MANTA and MANTA-Ray trials [18••], in which the sperm analysis of 120 patients (61 affected by ulcerative colitis, 5 by Crohn’ disease and 54 by inflammatory arthritis) was assesses (before and after Filgotinib). The MANTA-Ray is the first trial drawn for patients affected by inflammatory arthritis with the aim to assess the impact of a specific treatment on semen parameters.

To evaluate the effects of other tsDMARDs and other bDMARDs, it may be convenient that specific trials to assess the potential consequence of a therapy on male fertility will be planned.

Another missing link is the assessment of male fertility in PsA patients: only three studies specified the number of PsA patients included [15, 18••, 19••] with a very poor number of studied patients.

This topic is not of secondary importance, since it was shown that patients with psoriasis showed abnormal semen/sperm parameters and remarkably elevated leukocytes and values of seminal polymorphonuclear elastase, indicating a genital tract inflammation [66, 67].

Moreover, it was recently confirmed that a higher severity in psoriasis is associated with sexual dysfunction, hypogonadism, and abnormal sperm quality [68]. These latter points reinforce the idea that PsA targeted studies on male fertility are needed.

We also spotlight on varicocele risk in SpA: all data support a higher prevalence and incidence of varicocele in AS [12, 21], however clear data about varicocele in PsA and others SpA are lacking. It is still to be clarified if the presence of varicocele in AS and more in general in all SpA is associated with an impairment of sperm quality (as expected). In the same wavelength there are no univocal data on the fertility hormone axis in SpA [19••, 25].

Going deeper on the relationship between cytokines involved in SpA/AS and PsA and male fertility, there are several papers that showed as TNFa, particularly in high concentration has a toxic effect on spermatozoa [28–31]; instead, other cytokines and cellular pathway like IL-17, IL-12, IL-23 and JAK-STAT signal play a role in male fertility but the information are not entirely clear. However, studies that measure the cytokines concentration in sperm from SpA/AS and PsA patients have never been done. If the “cytokines semen profile” will be characterized in these patients, it could open some answers to several unresolved questions.

## Conclusion

The association between SpA, AS, PsA and male fertility impairment is an important unmet need. It seems that the experience of these inflammatory diseases at younger age could impair the family planning in a man life. Moreover, the relationship between disease activity and male infertility remains to be clarified, even if there are different studies that support a higher risk of semen quality impairment according with disease activity.

Among therapies, “milestones” used for SpA, AS and PsA, namely TNFi, showed a safety profile on human male fertility in clinical studies; recent evidence from prospective studies and clinical trials come for MTX and Filgotinib that showed a secure profile on semen quality.

To understand the impact of other therapies in these patients’ new clinical prospective studies are needed, even if there are not particular warning from the preclinical results.

## Data Availability

No datasets were generated or analysed during the current study.

## Declarations

### Disclaimer for Publishing Figure

All the authors certify that the paper contains 2 original figures.
